# Small bowel intussusception in adults

**DOI:** 10.1308/003588414X13824511650579

**Published:** 2014-01

**Authors:** J Potts, A Al Samaraee, A El-Hakeem

**Affiliations:** South Tyneside NHS Foundation Trust,UK

**Keywords:** Intussusception, Bowel obstruction, Bowel telescoping

## Abstract

Intussusception is the telescoping of a proximal segment of the gastrointestinal tract into an adjacent distal segment. This rare form of bowel obstruction occurs infrequently in adults. We report a case of small bowel intussusception in an adult male patient. We have also performed a literature review of this rare condition.

## Case history

A 50-year-old Caucasian man presented to the emergency department with a 7-week history of intermittent right upper quadrant and epigastric abdominal pain. He had visited his general practitioner, who arranged a stool test for *Helicobacter pylori*. This was found to be negative at a later stage. For few weeks before admission, the patient noticed that eating had exacerbated a cramp-like abdominal pain. However, he was managing his food up to two days prior to admission, when his abdominal symptoms worsened significantly. He became increasingly nauseated, belching a lot more than usual. His bowels had been working normally up to 48 hours before admission but stopped abruptly at this point. Despite this, he was still able to pass some flatus. He also vomited once in the emergency department.

His past medical history included previous peptic ulcer disease, hypertension, type 2 diabetes (diet controlled), a right hip replacement, accident related subdural haematoma (surgically evacuated at 37 years of age) and osteoarthritis. He had no history of abdominal surgery. His body mass index was >40kg/m^2^. He was a non-smoker and his alcohol intake was around 40–60 units a week. He had no relevant family history.

**Figure 1:**
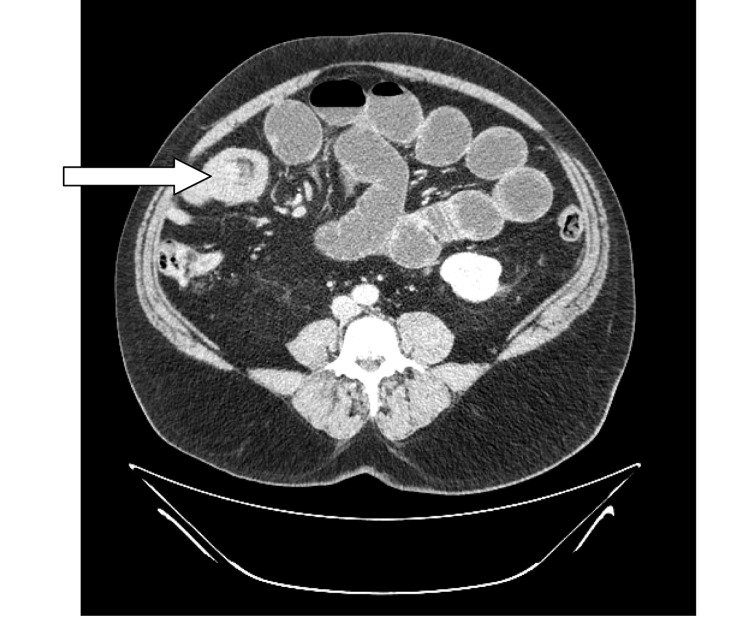
Axial computed tomography of the abdomen with contrast showing small bowel intussusception with ‘target’ lesion (arrow)

On admission, the patient was apyrexial, slightly tachycardic and normotensive. Abdominal examination revealed some distension with diffuse mild tenderness and exaggerated bowel sounds. No palpable hernia was felt during his abdominal examination. His routine blood tests (full blood count, renal and liver functions, and amylase) were all within the normal range. However, the C-reactive protein level was slightly raised at 22.4mg/l. His erect chest x-ray was essentially normal although the abdominal x-ray (AXR) showed dilated loops of small bowel with a maximum diameter of 5cm. He also underwent computed tomography (CT) of the abdomen and pelvis. This showed a small bowel intussusception (target lesion) as seen in Figure 1.

**Figure 2:**
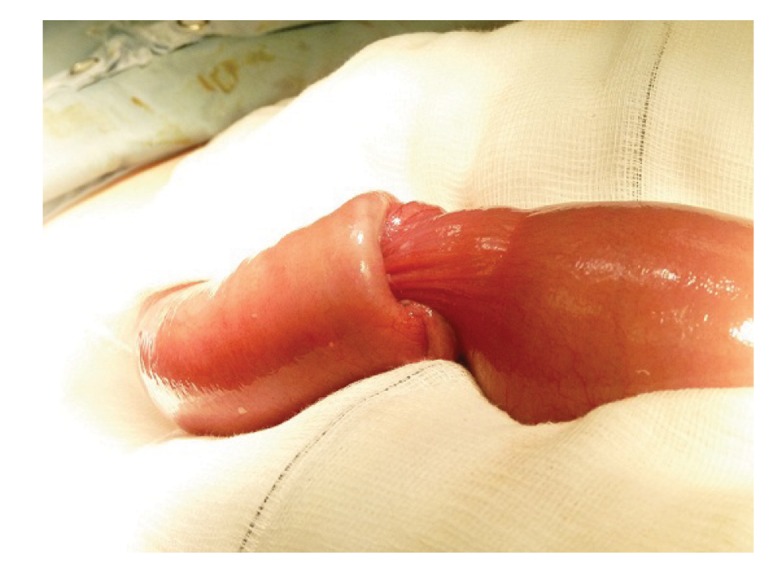
Resected intussuscepted small bowel

The patient subsequently went to theatre for a laparotomy, where a 6cm segment of non-gangrenous intussuscepted distal small bowel was found (ie enteroenteric intussusception), with a palpable polyp causing the lead point of the intussusception (Fig 2). No enlarged lymph nodes were felt in the mesentery of the affected small bowel. The intussuscepted small bowel segment was resected without any reduction attempts. This was followed by a side-to-side small bowel stapled anastomosis.

The postoperative period was generally uneventful apart from a simple wound infection that was treated successfully with the appropriate antibiotics. The patient made a steady recovery and went home a few days later. The histopathology report confirmed the picture of small bowel intussusception and showed that the polyp was of an inflammatory fibrous origin with no evidence of dysplasia or malignancy. The patient was followed up in the outpatient clinic few weeks later, when he was completely asymptomatic from the gastrointestinal point of view. Eventually, he was discharged completely from hospital follow-up.

## Methods for literature search

An electronic search of PubMed/MEDLINE®, Ovid® and Embase™ was performed, in addition to using the search engines Google/Google™ Scholar and Bing™. The search terms used were: ‘intussusception’, ‘bowel obstruction’ and ‘bowel telescoping’. Searches were screened and those studies thought to be relevant had full text versions retrieved. The references of all retrieved texts were searched for further relevant studies.

## Discussion

### Epidemiology

Bowel intussusception is the telescoping of a proximal segment of the gastrointestinal tract into an adjacent distal segment.^1^ It was first reported in 1674 by Barbette of Amsterdam.^2,3^ Two hundred years later, Sir Jonathan Hutchinson performed the first successful operation on a child with this condition in 1871.^4^

Intussusception can occur at any age but is most common in children between 5 and 10 months of age.^5^ The male-to-female ratio is approximately 3:1 in children whereas the prevalence is equal in adults.^6,7^ It is considered as the number one cause of bowel obstruction in children and the second most common cause of acute abdomen in children after appendicitis.^1^ Intussusception in adults is much less common, representing 5% of all intussusceptions, 1% of all bowel obstructions, 0.08% of all abdominal surgery and 0.003–0.02% of all hospital admissions.^6–9^ The overall incidence of intussusception in adulthood has been estimated to be around 2–3 cases/1,000,000 population/year.^3^

### Pathophysiology

Ninety-five per cent of intussusceptions in children are idiopathic^10^ whereas 80–90% of intussusceptions in adults have identifiable aetiology.^11^ Intussusception happens owing to invagination of one segment of the gastrointestinal tract and its mesentery (intussusceptum) into the lumen of an adjacent distal segment of the gastrointestinal tract (intussuscipiens). This may lead to lumen obstruction and ischaemia.^11^ The mechanism behind intussusception is not very clear but it could be explained by the presence of a lesion in the bowel wall or in its lumen that alters the normal peristalsis and serves as a lead point, which results in invagination of one segment of the bowel into the other.^12^ However, the cause of bowel intussusception without the presence of a lead point lesion is unknown.

Intussusception can be classified according to its location (enteroenteric, ileocolic, ileocaecal or colocolic) or according to aetiology (benign, malignant or idiopathic). Ninety per cent of intussusceptions in adults occur in the small or large bowel and the remaining ten per cent involve the stomach or surgically created stomas. The most common site is the small bowel while the least common types are coloanal and gastroduodenal intussusceptions.^13–15^

Renzulli and Candinas stated that 60% of small bowel intussusceptions in adults are caused by benign lesions.^16^ The remainder are caused by malignancy (30%) or are idiopathic (10%). Nevertheless, most colonic intussusceptions are caused by malignancy (60%).

Reported lesions or conditions that are associated with small bowel intussusception in adults include inflammatory fibrous polyps, lipomas, leiomyomas, haemangiomas, Meckel’s diverticula, metastatic lesions (from melanoma, breast and lung), leiomyosarcomas, malignant fibrous histiocytomas, lymphomas, carcinoid tumours, adenocarcinomas, Peutz-Jeghers syndrome, Henoch–Schönlein purpura, coeliac disease, Crohn’s disease, strictures, lymphadenitis and human immunodeficiency virus related infections/malignancies. Trauma and operative factors (eg anastomosis sites, adhesions, suture lines and feeding jejunostomy) are also associated with small bowel intussusception in adults.^3,17,18^ In the paediatric population, intussusception is most commonly associated with hyperplasia of Peyer’s patches secondary to viral infections.^7^

### Clinical presentation

The symptoms in adults are usually chronic and depend on the site of the intussusception. Despite this, patients with relatively short history presentations (like in our case report) have also been described although these are much less common. Intermittent attacks of non-specific abdominal pain (with or without bowel obstruction) seem to be the most common presenting symptom. Other associated symptoms include nausea, vomiting and rectal bleeding. In addition, the finding of a clinically palpable abdominal mass has been reported with various rates in the literature. Other associated symptoms such as weight loss and constipation may indicate the presence of an associated serious underlying pathology (eg malignancy). In children, the triad of abdominal pain, a palpable sausage-shaped abdominal mass and red jelly-like stool is a classic presentation of small bowel intussusception. However, this triad is rarely seen in adults.^5,13,19^

### Diagnosis

Owing to its rarity and non-specific elusive presentations, the clinical diagnosis of intussusception in adults is often delayed and challenging. Various imaging modalities have been used to help in establishing the diagnosis. Still, the diagnosis is frequently confirmed only during surgical intervention.

CT of the abdomen seems to be the radiological investigation of choice, with a sensitivity of 71.4–87.5% and a specificity of 100% in the prospect of diagnosis of intussusception.^3^ Moreover, CT has been used widely in various clinical scenarios. This has increased the detection rates of incidental gastrointestinal pathologies such as intussusception in adults.^20^

Depending on the axial projection, abdominal CT findings that might indicate the presence of small bowel intussusception include the ‘target’ or ‘doughnut’ signs as well as a sausage-shaped mass or pitchfork image. Obviously, these findings should be correlated with the patient’s clinical picture before making the final diagnosis. Moreover, abdominal CT helps in identification of the lead point lesions when present and other associated pathologies such as metastatic malignancies.^20,21^

Kim *et al* stated: ‘*At abdominal CT, the presence of a bowel-within-bowel configuration with or without mesenteric fat and mesenteric vessels is pathognomonic for intussusception*. […] *CT can be helpful in distinguishing between lead point intussusception and non-lead point intussusception and has the potential to reduce the prevalence of unnecessary surgery*. ’^20^

Lvoff *et al* performed an interesting study to investigate whether abdominal CT can be used to distinguish self-limiting cases of adult small bowel intussusception from those requiring surgery.^22^ They concluded that an intussusception that is shorter than 3.5cm is likely to be self-limiting. Nevertheless, the study was retrospective in a single centre and there was a lack of pathological correlation.

Abdominal ultrasonography can also be used to check for small bowel intussusception in adults and children. The classical ultrasonography findings include ‘target’ or ‘doughnut’ signs on transverse view, the ‘pseudokidney’ sign on oblique view and the ‘trident’ sign on longitudinal view.^3,23–25^ Ultrasonography carries no radiation risk. However, it is operator dependent. Furthermore, gas in distended loops of bowel and the patient’s body habitus (obesity) might affect the quality of the views and, consequently, the radiological findings. It is therefore more useful in children and perhaps thin adults.

Plain AXR is usually considered to rule out bowel obstruction in the emergency setting. In cases with small bowel intussusception, AXR might show signs of bowel obstruction such as dilated loops of bowel or fluid level in the bowel lumen, and rarely a mass lesion or intraluminal air trapped between the walls of the intussusceptum and intussuscipiens (air crescent sign). These findings nevertheless lack the specificity and sensitivity to diagnose intussusception.^3,26^

Barium studies have also been reported to diagnose bowel intussusception in adult patients with long-term nonspecific abdominal pain. However, these studies are contraindicated in patients suspected to have bowel obstruction owing to the risk of perforation.^27^ In addition, endoscopic approaches such as enteroscopy, capsule endoscopy and colonoscopy have been used in establishing the diagnosis in elective cases.^3^

### Treatment

Unlike the paediatric population, reduction of the intussuscepted bowel with barium or air is not indicated in adults. This is due to the significant rate of other pathologies associated with bowel intussusception in adults.^12^ As a result, bowel intussusception in adults is a condition that commonly warrants surgical intervention.

The high incidence of malignancy associated with colonic intussusception perhaps justifies performing a primary oncological resection of the affected bowel without reduction attempts. Reduction carries risks of perforation and the theoretical possibility of tumour seeding.^28^

The fact that the incidence of malignancy associated with small bowel intussusception is less common than with the large bowel has resulted in a debate on whether to attempt reducing the intussuscepted small bowel before resection to save the small bowel length.^13^ From our experience with the reported case, it was difficult to decide for sure whether the associated lead point lesion (polyp) was benign or malignant macroscopically.

The accessibility of intraoperative histopathological tissue diagnosis facilities can also help in determining the extent of surgical resection. However, such facility is not always accessible, keeping in mind that emergency operations are necessary in up to 60% of all adult patients with intussusception.^11^

Some authors advise that simple reduction is acceptable in post-traumatic or idiopathic intussusceptions, where no pathological cause could be identified, obviously after the exclusion of bowel ischaemia or perforation.^13,29,30^

Patients with multiple small intestinal polyps like those in Peutz-Jeghers syndrome are liable to have frequent intussusceptions. In such scenarios, a combined approach of limited intestinal resections and multiple snare polypectomies is advised to avoid developing short bowel syndrome.^12,31^

In summary, small bowel intussusception in adults commonly requires surgical resection of the affected bowel. The choice of the surgical approach (open or laparoscopic) usually depends on the patient’s clinical condition, the extent and level of the intussusception, and the availability of local resources and expertise. Preoperative tissue diagnosis of the lead point lesion helps in performing limited resection in benign conditions. However, this is not always available as the diagnosis of intussusception is frequently confirmed during surgical intervention.

## Conclusions

Small bowel intussusception in adults is rare and often challenging to diagnose owing the elusive, non-specific symptoms. A high index of clinical suspicion combined with the appropriate imaging might help in establishing an early diagnosis, excluding any associated malignancies and avoiding serious complications like perforation and peritonitis. Abdominal CT seems to be the radiological investigation of choice, with its high sensitivity and specificity in this prospect. Nevertheless, the diagnosis is made frequently on the operating table. In the presence of a lead point lesion but no preoperative tissue diagnosis, surgical intervention in the form of bowel resection without reduction is advisable.

## References

[1] Agha FP. Intussusception in adults. *Am J Roentgenol* 1986; : 527–531. 3484870 10.2214/ajr.146.3.527

[2] Barbette de Moulin D. Paul, MD: a seventeenth-century Amsterdam author of best-selling textbooks. *Bull Hist Med* 1985; : 506–514. 3912022

[3] Manouras A, Lagoudianakis EE, Dardamanis D *et al* Lipoma induced jejunojejunal intussusception. *World J Gastroenterol* 2007; : 3,641–3,644. 10.3748/wjg.v13.i26.3641 PMC4146808 17659719

[4] Sarma D, Prabhu R, Rodrigues G. Adult intussusception: a six-year experience at a single center. *Ann Gastroenterol* 2012; : 128–132. 24714146 PMC3959399

[5] Intussusception. Medscape reference. http://emedicine.medscape.com/article/930708-overview#a0101 (cited 4 2013).

[6] Eisen LK, Cunningham JD, Aufses AH. Intussusception in adults: institutional review. *J Am Coll Surg* 1999; : 390–395. 10195723 10.1016/s1072-7515(98)00331-7

[7] Al-Saad S, Al-Helo H. Ileocolic intestinal intussusception in adults: a case report. *Bahrain Med Bull* 2003; : 43–45.

[8] Azar T, Berger DL. Adult intussusception. *Ann Surg* 1997; : 134–138. 10.1097/00000658-199708000-00003 PMC1190946 9296505

[9] Huang WS, Changchien CS, Lu SN. Adult intussusception: a 12-year experience, with emphasis on etiology and analysis of risk factors. *Chang Gung Med J* 2000; : 284–290. 10916229

[10] Mandal S, Kawatra V, Dhingra KK *et al* Lipomatous polyp presenting with intestinal intussusception in adults: report of four cases. *Gastroenterol Res* 2010; : 229–231. 10.4021/gr232e PMC5139722 27957003

[11] Paskauskas S, Pavalkis D. Adult Intussusception. In: Lule G. *Current Concepts in Colonic Disorders*. Rijeka, Croatia: InTech; 2012 pp 1–22.

[12] Marinis A, Yiallourou A, Samanides L *et al* Intussusception of the bowel in adults: a review. *World J Gastroenterol* 2009; : 407–411. 19152443 10.3748/wjg.15.407 PMC2653360

[13] Yalamarthi S, Smith RC. Adult intussusception: case reports and review of literature. *Postgrad Med J* 2005; : 174–177. 15749793 10.1136/pgmj.2004.022749 PMC1743231

[14] Stubenbord WT, Thorbjarnarson B. Intussusception in adults. *Ann Surg* 1970; : 306–310. 5433296 10.1097/00000658-197008000-00019 PMC1397058

[15] Nesbakken A, Haffner J. Colo-recto-anal intussusception. Case report. *Acta Chir Scand* 1989; : 201–204. 2741629

[16] Renzulli P, Candinas D. Idiopathic small-bowel intussusception in an adult. *CMAJ* 2010; : E148. 19969561 10.1503/cmaj.090457 PMC2826481

[17] Pavlidis TE, Atmatzidis KS, Galatianos I *et al* Intestinal intussusception in adults – our experience and review of the literature. *Ann Gastroenterol* 2004; : 75–78.

[18] Duijff JW, van der Burg B, Aarts NJ *et al* Intussusception in adults: report of four cases and review of the literature. *Case Rep Gastroenterol* 2007; : 59–64. 21487473 10.1159/000107473 PMC3073789

[19] Yakan S, Caliskan C, Makay O *et al* Intussusception in adults: clinical characteristics, diagnosis and operative strategies. *World J Gastroenterol* 2009; : 1,985–1,989. 19399931 10.3748/wjg.15.1985 PMC2675089

[20] Kim YH, Blake MA, Harisinghani MG *et al* Adult intestinal intussusception: CT appearances and identification of a causative lead point. *Radiographics* 2006; : 733–744. 16702451 10.1148/rg.263055100

[21] Spiridis C, Kambaroudis A, Ntinas A *et al* Intussusception of the small bowel secondary to malignant metastases in two 80-year-old people: a case series. *J Med Case Rep* 2011; : 176. 21569293 10.1186/1752-1947-5-176 PMC3113992

[22] Lvoff N, Breiman RS, Coakley FV *et al* Distinguishing features of self-limiting adult small-bowel intussusception identified at CT. *Radiology* 2003; : 68–72. 12668740 10.1148/radiol.2272020455

[23] Boyle MJ, Arkell LJ, Williams JT. Ultrasonic diagnosis of adult intussusception. *Am J Gastroenterol* 1993; : 617–618. 8470658

[24] Alessi V, Salerno G. The ‘hay-fork’ sign in the ultrasonographic diagnosis of intussusception. *Gastrointest Radiol* 1985; : 177–179. 3888768 10.1007/BF01893097

[25] Anderson DR. The pseudokidney sign. *Radiology* 1999; : 395–397. 10.1148/radiology.211.2.r99ma21395 10228519

[26] White SJ, Blane CE. Intussusception: additional observations on the plain radiograph. *Am J Roentgenol* 1982; : 511–513. 6981315 10.2214/ajr.139.3.511

[27] Takeuchi K, Tsuzuki Y, Ando T *et al* The diagnosis and treatment of adult intussusception. *J Clin Gastroenterol* 2003; : 18–21. 12488701 10.1097/00004836-200301000-00007

[28] Udeh EI, Amu CO, Dakum NK *et al* An atypical presentation of adult ileocolic intussusception. *Open Access Surg* 2010; : 143–145.

[29] Begos DG, Sandor A, Modlin IM. The diagnosis and management of adult intussusception. *Am J Surg* 1997; : 88–94. 9074370 10.1016/S0002-9610(96)00419-9

[30] Kitamura K, Kitagawa S, Mori M, Haraguchi Y. Endoscopic correction of intussusception and removal of a colonic lipoma. *Gastrointest Endosc* 1990; : 509–511. 2227328 10.1016/s0016-5107(90)71128-5

[31] Lin BC, Lien JM, Chen RJ *et al* Combined endoscopic and surgical treatment for the polyposis of Peutz-Jeghers syndrome. *Surg Endosc* 2000; : 1,185–1,187. 11148795 10.1007/s004640000029

